# Exercise intensities modulate cognitive function in spontaneously hypertensive rats through oxidative mediated synaptic plasticity in hippocampus

**DOI:** 10.1111/jcmm.16816

**Published:** 2021-07-30

**Authors:** Cheng‐Che Lee, De‐Yu Wu, Syue‐yi Chen, Yi‐Pin Lin, Tsung‐Ming Lee

**Affiliations:** ^1^ Kang‐Ming Senior High School Tainan Taiwan; ^2^ Catholic Sheng Kung Girls’ High School Tainan Taiwan; ^3^ Cardiovascular Institute An Nan Hospital China Medical University Tainan Taiwan; ^4^ Department of Neurology An Nan Hospital China Medical University Tainan Taiwan; ^5^ Department of Medicine China Medical University Taichung Taiwan

**Keywords:** exercise, Morris water maze, reactive oxygen species, spontaneously hypertensive rats, synaptic plasticity

## Abstract

Oxidative damage in the brain may lead to cognitive impairments. There was considerable debate regarding the beneficial effects of physical exercise on cognitive functions because exercise protocols have varied widely across studies. We investigated whether different exercise intensities alter performance on cognitive tasks. The experiment was performed on spontaneously hypertensive rats (6 months at the established phase of hypertension) distributed into 3 groups: sedentary, low‐intensity exercise and high‐intensity exercise. Systolic blood pressure measurements confirmed hypertension in spontaneously hypertensive rats. In comparison to normotensive Wistar‐Kyoto rats, sedentary spontaneously hypertensive rats had similar escape latencies and a similar preference for the correct quadrant in the probe trial. Compared to the sedentary group, the low‐intensity exercise group had significantly better improvements in spatial memory assessed by Morris water maze. Low‐intensity exercise was associated with attenuated reactive oxygen species, as measured by dihydroethidine fluorescence and nitrotyrosine staining in the dentate gyrus of the hippocampus. This was coupled with increased numbers of neurons and dendritic spines as well as a significant upregulation of synaptic density. In contrast, the beneficial effects of low‐intensity exercise are abolished in high‐intensity exercise as shown by increased free radical levels and an impairment in spatial memory. We concluded that exercise is an effective strategy to improve spatial memory in spontaneously hypertensive rats even at an established phase of hypertension. Low‐intensity exercise exhibited better improvement on cognitive deficits than high‐intensity exercise by attenuating free radical levels and improving downstream synaptic plasticity.

## 1 INTRODUCTION

Hypertension and hypertension‐related diseases should be a top public health concern. Hypertensive adults have been associated with a high prevalence of cognitive impairment such as spatial disorientation,^1^ resulting in a worse quality of life. Spontaneously hypertensive rats (SHR) are frequently used as a genetic model of hypertension to study human hypertension. In these models, the age‐dependent increases in arterial blood pressure and pathological changes in brain are somewhat similar to the damage caused to the brain by hypertension in humans.^2^

Brain is an organ very sensitive to oxidative stress. Reactive oxygen species (ROS) production might have neurotoxic effects for brain plasticity. ROS such as superoxide has been shown to be increased in SHR.^3^ ROS caused by hypertension accumulates in different organs, which may persistently destroy the cells and lead to diseases. The brain has been shown to be at greater vulnerability to ROS‐induced oxidative stress due to the lack of antioxidative enzymes^4^ and as it consumes 20% of the body's oxygen supply.^5^ Endogenous antioxidants were downregulated in SHR brain.^6^ The precise chain of ROS‐mediated cognitive decline within the central nervous system is an interesting topic. The hippocampus plays a key role in learning and memory, and it has been demonstrated to be the most susceptible area of the brain to oxidative stress^7^, ^8^ and therefore to be at the highest risk of functional decline. Furthermore, dentate gyrus granule cells, which receive sensory information from fibres of the perforant pathway, have also been shown to be prone to oxidative stress.^9^ Region‐specific elevation of ROS within dentate gyrus is important and can have significant functional consequences. Previous studies have shown that dendritic retraction in the dentate gyrus has traditionally corresponded to hippocampus‐dependent spatial memory deficits.^10^ ROS are considered to be toxic, causing oxidation of membrane lipids, damage to nucleic acids, changes in protein conformation and deficits in synaptic plasticity. Rats subjected to high ROS showed marked deficits in learning and memory functions.^11^ Exogenous administration of superoxide results in transient reduction in postsynaptic response, followed by impaired long‐term potentiation, which can be rescued by superoxide dismutase administration.^12^

It is generally acknowledged that synapses enable the transmission of chemical and electrical messages from neurons to other cells and that synaptic plasticity plays an important neurobiological role in learning and memory ability.^13^ Many studies have demonstrated an association between reduced synaptic plasticity and impaired learning and memory.^14^, ^15^ Synaptophysin has been shown to be expressed at presynaptic terminals, and it has been shown to be involved in the formation of synapses and release of neurotransmitters.^16^, ^17^ Furthermore, it has been demonstrated to be a reliable marker of synaptic plasticity in the brain, and it has been widely used for qualitative and quantitative analysis of synaptic density.^16^ The mice with synaptophysin knockout have been shown to be associated with impaired hippocampal integrity which leads to deficits in learning and memory.^18^ Antioxidants have been to attenuate ROS‐mediated reduction of synaptophysin in the brain.^19^

Epidemiological studies showed that physical exercise has been proposed as a strategy that improves learning and memory.^20^ Adult hippocampal neurogenesis is tightly regulated by physical exercise.^21^ Low physical activity was found to be a major risk factor for cognitive decline.^22^ Exercise training yielded protection against cognitive decline in the hippocampus of hypertensive rats.^23^ However, there were contradictory effects of physical exercise on cognitive function which depend on the intensity of physical activity as well as the type of memory evaluated. Different exercise intensities may affect effects on learning and memory differentially. Exercise has been shown to enhance the recognition of objects in animals categorized as being low‐runners, but to compromise performance in those categorized as being high‐ and very‐high runners.^24^ Appropriate exercise intensity can benefit health by inducing a strengthening in the antioxidant defence system.^25^ However, exhaustive exercise can generate excessive ROS, leading to ROS‐mediated tissue damages.^25^ Systemic ROS induced the breakdown of the brain‐blood barrier which provides a cycle of damage by increasing the expression of a number of adhesion molecules.^26^ Exercise capacity declines with age, and thus, older animals may not be able to sustain the exercise intensity used for training adaptation in young animals; hence, alternative reduced intensity exercise programmes are suggested for ageing animals. However, the beneficial effects of the alternative exercise programmes on cognitive functions remained unknown. Therefore, in the current study, we investigated whether low‐intensity physical exercise is an effective strategy to enhance learning and memory in rodents. This schedule of exercise training (5 days per week, 30 min per session at low and high intensity, for 4 weeks) was chosen to mimic exercise conditions in humans.^27^ We assessed the effects of (1) hypertension, with 24‐week‐old male SHR and Wistar‐Kyoto (WKY) rats, (2) exercise, with low and high intensity, on cognitive function in SHR, and (3) the role of ROS and synaptophysin in mediating cognitive function.

## 2 MATERIAL AND METHODS

### 2.1 Ethics

All experiments were conducted according to the NIH guidelines for the care and use of animals. In addition, the animal protocols were approved by the Institutional Animal Care and Use Committees at the Chi‐Mei Hospital (Protocol no. 21/2009). Male rats (3 per group) were housed in cages at a constant temperature (22 ± 1°C) and humidity (60% ± 10%) with a 12‐h light and12‐h dark cycle. Access to food and water was allowed *ad libitum* and in a 12‐h light:12‐h dark cycle.

### 2.2 Animals

The experiments were performed on male SHR of 24‐week‐old and normotensive age‐matched control WKY, all purchased from LASCO (Taipei, Taiwan). Animal age of 24‐week‐old corresponded to adult and ‘middle‐aged’ humans^28^ as well as represented the periods of sustained/chronic hypertension. Systolic blood pressure (BP) was measured monthly from week 8 to 24 in conscious rats by tail plethysmography (BP‐98A, Softron Beijing). To clarify the importance of exercise intensity, the rats are submitted to exercise with overloads tied to the thorax with a battery equal to about 8% of body weight, viewed as high‐intensity exercise (HIE).^29^ Swimming without burden implies low‐intensity exercise (LIE). Thus, rats were divided into 4 groups (6 animals per group) as follows: WKY sedentary (WKY), SHR sedentary (Sed SHR), SHR with LIE and SHR with HIE.

### 2.3 Swimming exercise protocol

The animals were allowed to acclimatize before the experiments by placing them in a tank containing 1.5 cm water at 25 ± 1°C for 2 min. This procedure was important to avoid causing the animals stress, especially at the beginning of the exercise protocol. The swimming exercise training in a plastic tank 150 × 60 × 50 cm (length × width × depth) with the water temperature kept at 25 ± 1°C was used. Before beginning experimental processes, the rats had an acclimation period of four days, which involved making the rats swim for 5–10 min, and then gradually increasing the training period to 30 min per day. Rats were trained to swim 30 min per day, 5 days a week, between 8 a.m. and 11 a.m. during 4 weeks. Sedentary rats were placed in shallow water to stand normally with their head above the water without need to swim, 30 min., 5 days per week.

Hippocampal‐dependent spatial learning and memory were evaluated using the Morris water maze with a training trial and a probe trial.^30^ The training trial stage was conducted on days 1–4 and consisted of four trials lasting for a maximum of 90 s with an inter‐trial interval of 30 s. The rats were allowed to rest on a platform for 30 s regardless of whether or not the trial was successful. A researcher guided the rats to find the platform if they could not find it within 90 s. The rats were then made to stay on the platform for 30 s, and the escape latency was recorded as 90 s. The time taken (escape latency) to reach the platform and the distance covered were measured, and these parameters were used to assess spatial learning. The probe trial is a standard feature of Morris water maze in which the platform is removed 24 h after a session of spatial learning. The percentage of time that the rats were in the training quadrant during the probe trial was recorded and interpreted as spatial memory.

The behaviour of the rats was recorded using a camera aligned perpendicularly over the centre of the pool. The camera was connected to a monitor, a videorecorder and a contrast‐sensitive videotracker (GERIN Technology Co., Taiwan) to track the swimming paths of the rats. We used computer programmes to determine speed of movement. Videotracker data were recorded on a personal computer using customized software.

### 2.4 Histological staining

At completion of Morris water maze, all groups were killed by decapitation and their brains were dissected on ice to remove the hippocampus. The tissues were embedded in paraffin and a series of slides were sampled.

Nissl staining, a useful marker for the neuron state, was used to stain nucleic acid. The cell layer of the dentate gyrus region was captured using a digital microscope camera. Positively Nissl‐stained cells were expressed as the number of cells per area selected in the region of interest.

Dendritic spine density in the dentate gyrus was evaluated using Golgi staining with a Hito Golgi–Cox OptimStain PreKit according to the manufacturer's instructions (Hitobiotec, Kingsport) and recorded as the number of spines per micrometre. The number of positively stained dendritic spines was counted in a blinded manner using Image‐Pro Plus software (Media Cybernetics, Silver Spring).

To determine the numbers of presynaptic and postsynaptic puncta, we measured synaptophysin and postsynaptic density protein 95 (PSD95) intensities by immunohistochemical analysis. Hippocampal dentate gyrus sections were incubated with 5% goat serum to block non‐specific binding, followed by incubation overnight with anti‐PSD95 antibodies (1:200, Abcam, Cambridge) and rabbit anti‐synaptophysin antibodies (1:200, Abcam, Cambridge) at 4˚C.

### 2.5 In situ detection of superoxide anion and nitrotyrosine

Production of intracellular superoxide in the dentate gyrus was then evaluated using in situ dihydroethidium (DHE) fluorescence (1 µM, Invitrogen‐Molecular Probes, Eugene). Paraffin‐embedded tissues (5 µm) were incubated for 30 min at room temperature with DHE in PBS (10 mM) in a humidified dark container. Superoxide radicals in nuclei were converted to the red fluorescence compound ethidium, and the density of the images was recorded as arbitrary units per millimetre square field.

Nitrative stress was evaluated through the detection of nitrotyrosine, a biomarker for the formation of peroxynitrite, a by‐product of ^•^NO and O_2_^•−^, by immunohistochemistry in hippocampal dentate gyrus sections. After antigen retrieval and endogenous peroxidase quenching, immunostaining for nitrotyrosine was performed using rabbit polyclonal nitrotyrosine antibodies (1:200, Millipore, Bedford) overnight at 4°C.

### 2.6 Western blot of synaptophysin and PSD95

There is convincing evidence that tyrosine phosphorylation of synaptophysin is required for long‐term potentiation, a cellular correlate of learning and memory.^31^ Rat hippocampus was dissected after snap frozen. Total protein fractions in the supernatant were obtained by vigorously mixing the homogenates for 20 min on a rotator at 4°C followed by centrifugation at 12,000 *g* for 30 min. The total concentration of protein was then measured using Protein Assay Dye Reagent Concentrate (Bio‐Rad). Western blots were performed and analysed as previously described.^32^ Antibodies used for Western blots were as follows: synaptophysin (Abcam, Cambridge) and rabbit PSD95 antibody (Abcam). β‐actin (Cell Signaling Technology) was used as a loading control for hippocampus lysates.

### 2.7 Laboratory measurement

The reaction of nitro blue tetrazolium in TRIS buffer was used to measure the plasma level of superoxide at a wavelength of 530 nm. This reaction is superoxide radical‐specific as inhibition of the reduction can be achieved by superoxide dismutase.^33^

We assessed lipid peroxidation levels in the hippocampus by measuring the thiobarbituric‐acid‐reacting substances (TBARS) in homogenates, as previously described.^34^ The hippocampal samples were mixed with 1 ml 10% trichloroacetic acid and 1 ml 0.67% thiobarbituric acid. They were then heated in a boiling water bath for 15 min, and butanol was added to the solution. After centrifugation, thiobarbituric‐acid‐reacting substances were determined from the absorbance at 535 nm. The results above were expressed as nmol of malondialdehyde (MDA)/g wet tissue.

### 2.8 Statistical analysis

All values are expressed as the mean ± SD except Table 1. Data derived from the body weight, blood pressure and water maze experiments were analysed using a two‐way repeated measures ANOVA with group and time as factors followed by Bonferroni's multiple comparisons post hoc test to evaluate the statistical significance between groups. Data for histological staining, superoxide anion, nitrotyrosine, Western blot and MDA concentrations were evaluated by one‐way ANOVA and post hoc test. The analyses were performed with the (SPSS) for Windows. Differences were considered to be statistically significant at a *p* value of < 0.05.

**TABLE 1:** Haemodynamics and body weights at the end of study

Parameters	WKY	Sed SHR	LIE SHR	HIE SHR
*n*	6	6	6	6
Heart rate, bpm	372 ± 16	358 ± 25	349 ± 13	381 ± 23
SBP, mm Hg	113 ± 3	172 ± 8***	166 ± 5***	180 ± 6***
DBP, mm Hg	93 ± 2	124 ± 8**	122 ± 5**	125 ± 5**
BW, gm	604 ± 12	365 ± 5***	341 ± 8***	336 ± 8***

Note

Values are mean ± SEM. **p < 0.01, ***p < 0.001 compared with WKY.

Abbreviation: BW, body weight; DBP, diastolic blood pressure; HIE, high‐intensity exercise; LIE, low‐intensity exercise; SBP, systolic blood pressure; Sed, sedentary.

## 3 RESULTS

Wistar‐Kyoto significantly outweighed three groups of SHR (Table 1). The effect of exercise training on physical characteristics is presented in Table 1. The rats in the LIE did not have a significant difference of body weight compared with age‐matched sedentary SHR. There were no significant differences in body weight between the two exercise groups at baseline and the end of the study.

**FIGURE 1:**
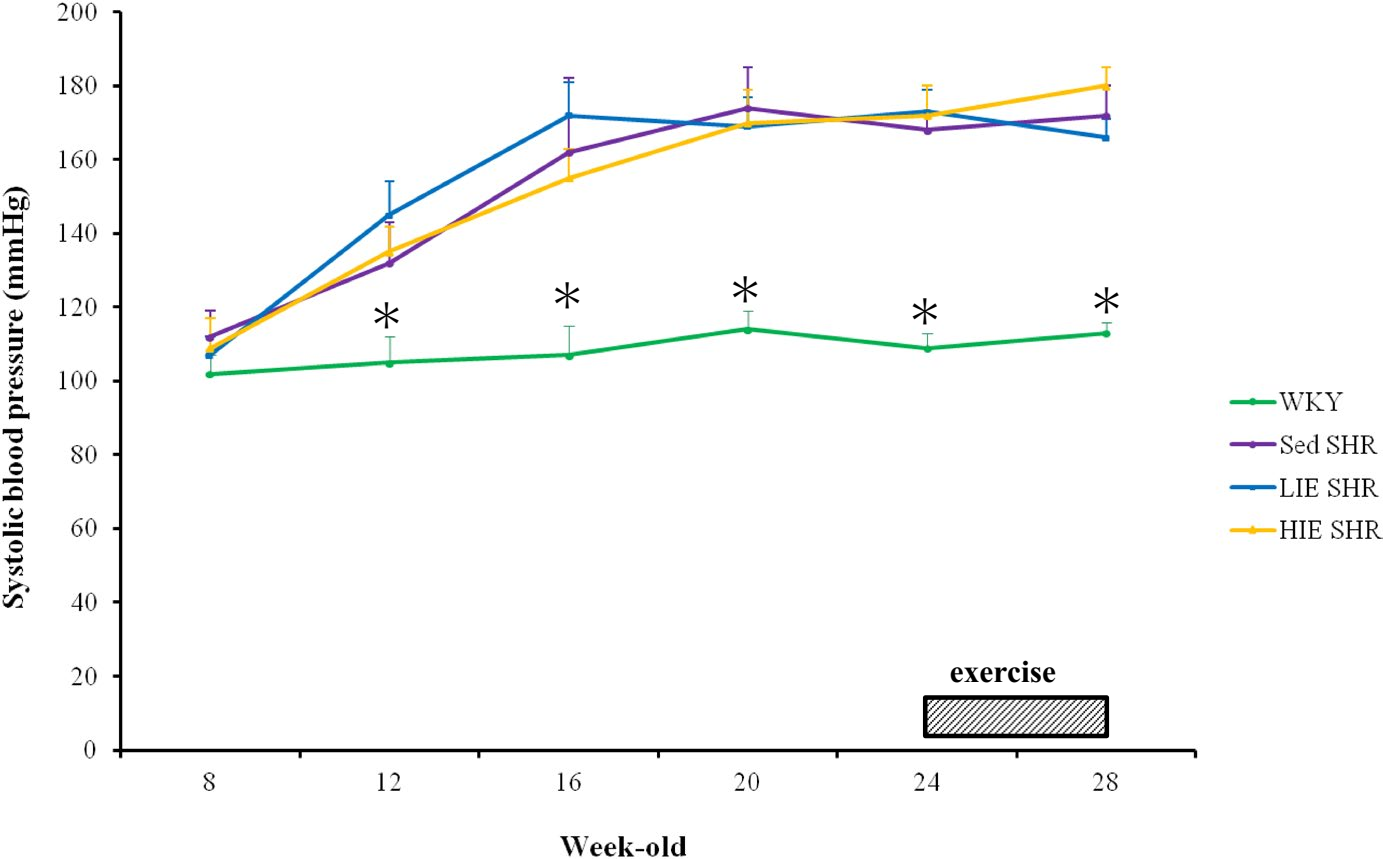
Time course in tail SBP of WKY and SHR. Systolic blood pressure was significantly higher in SHR at 12 weeks of age and gradually increased thereafter. Values are expressed as means ± SD (*n* = 6 per group) and were analysed by repeated measures ANOVA with group and time as factors followed by Bonferroni's multiple comparisons post hoc test. *p < 0.05 versus Sed SHR, LIE SHR and HIE SHR at the same age

Tail‐cuff systolic pressures measured in SHR and WKY are shown in Figure 1. There was a significant increase in systolic BP in the SHR groups from 12 weeks of age onwards, with a further increase at 18 weeks. The blood pressures among the three SHR groups were similar, independent of exercise training. In contrast, there was no significant variation in systolic BP in the WKY group during the study period.

### 3.1 Effects of hypertension and exercise intensity on systemic and hippocampal RONS levels

**FIGURE 2:**
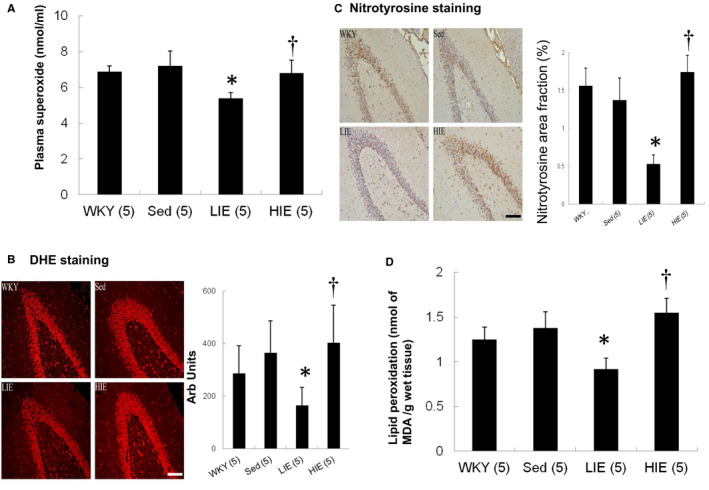
Superoxide and nitrotyrosine levels. (A) Plasma superoxide levels, (B) hippocampal DHE staining as an index of superoxide stress and quantitative analysis, (C) hippocampal nitrotyrosine immunoreactive staining and quantitative analysis and (D) hippocampal TBARS to measure the lipid peroxidation level. Hippocampal DHE (red fluorescent) and nitrotyrosine (brown) staining showed less intense signals (nuclear position for DHE and cytoplasm for nitrotyrosine) in LIE SHR. Bar = 100 μm. Data expressed as mean  ± SD and analysed by one‐way ANOVA followed by Bonferroni's *post hoc* test. *p < 0.05 versus WKY and sed SHR; ^†^ *p* < 0.05 versus LIE SHR

Plasma superoxide levels were measured to evaluate the systemic effect of exercise on levels of circulating ROS. Plasma superoxide levels in LIE SHR were significantly reduced compared with sedentary and HIE SHR (5.41 ± 0.32 vs. 7.19 ± 0.85 in sedentary SHR and 6.82 ± 0.72 nmol/ml in HIE SHR, *p *< 0.05, Figure 2A).

The DHE reaction was used to evaluate the levels of ROS generated in the hippocampus, as this reaction can detect the ROS superoxide anion in tissue (Figure 2B). There was similar fluorescent signal between WKY and sedentary SHR. Compared with sedentary SHR, LIE SHR had significantly decreased intensity of the fluorescent signal. Hippocampal superoxide in HIE SHR was significantly increased compared with LIE SHR.

Similarly, hippocampal nitrotyrosine in LIE SHR significantly decreased as compared to HIE SHR (*p *< 0.01, Figure 2C).

To further confirm hippocampal lipid peroxidation levels, we measured TBARS. Figure 2D showed a significant increase of 68% in hippocampus of HIE SHR in lipid peroxidation level compared with LIE SHR (*p *< 0.01).

### 3.2 Effects of variable exercise intensity on the neurons and spines in the hippocampus

**FIGURE 3:**
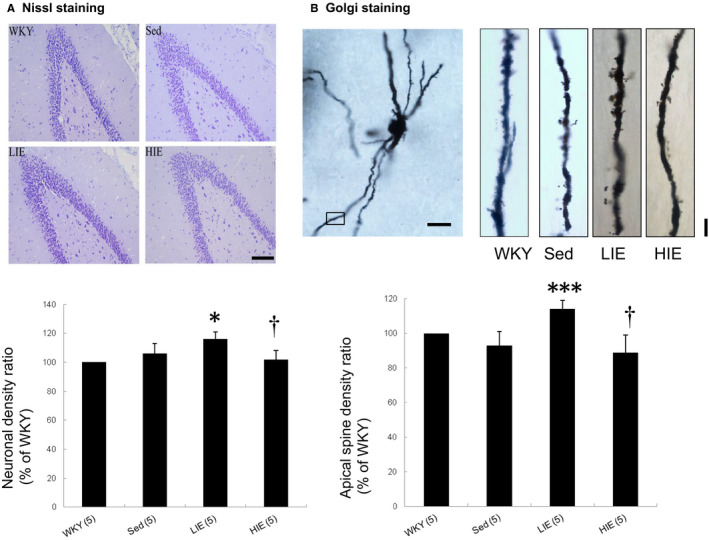
Nissl staining and Golgi stain. (A) Nissl staining of the cells in the dentate gyrus region showing improvement in the Nissl granules and darker staining with more ribosomes inside in the LIE group compared to sedentary SHR. Scale Bar = 100 μm. (B) Golgi stain. Representative single granule neuron morphology with Golgi staining and the framed apical dendritic section for spine density analysis (Scale Bar = 50 μm). LIE increases the number of dendritic apical spines in dentate gyrus. Scale Bar = 5 μm. Data expressed as mean  ± SD and analysed by one‐way ANOVA followed by Bonferroni's *post hoc* test. *p < 0.05, ***p < 0.001 versus WKY and sed SHR; ^†^ *p* < 0.05 versus LIE SHR

To clarify the synaptic plasticity changes, we investigated the hippocampal neuronal and dendritic structures. The total number of surviving neurons in the hippocampal dentate gyrus region was similarly as shown by Nissl staining between WKY and sedentary SHR (Figure 3A). Compared with HIE, rats in LIE had significantly increased neuronal cells survival in the dentate gyrus region of the hippocampus.

We next examined dendritic spine formation in the dentate gyrus by using Golgi staining to visualize spine morphology (Figure 3B). Mean spine density was significantly increased by 28.3 ± 1.8% (*p* < 0.001) in the dentate gyrus of LIE SHR compared with sedentary SHR. However, HIE decreased dendritic spine density compared with LIE.

### 3.3 Effects of variable exercise intensity on synapse number in the hippocampus

**FIGURE 4:**
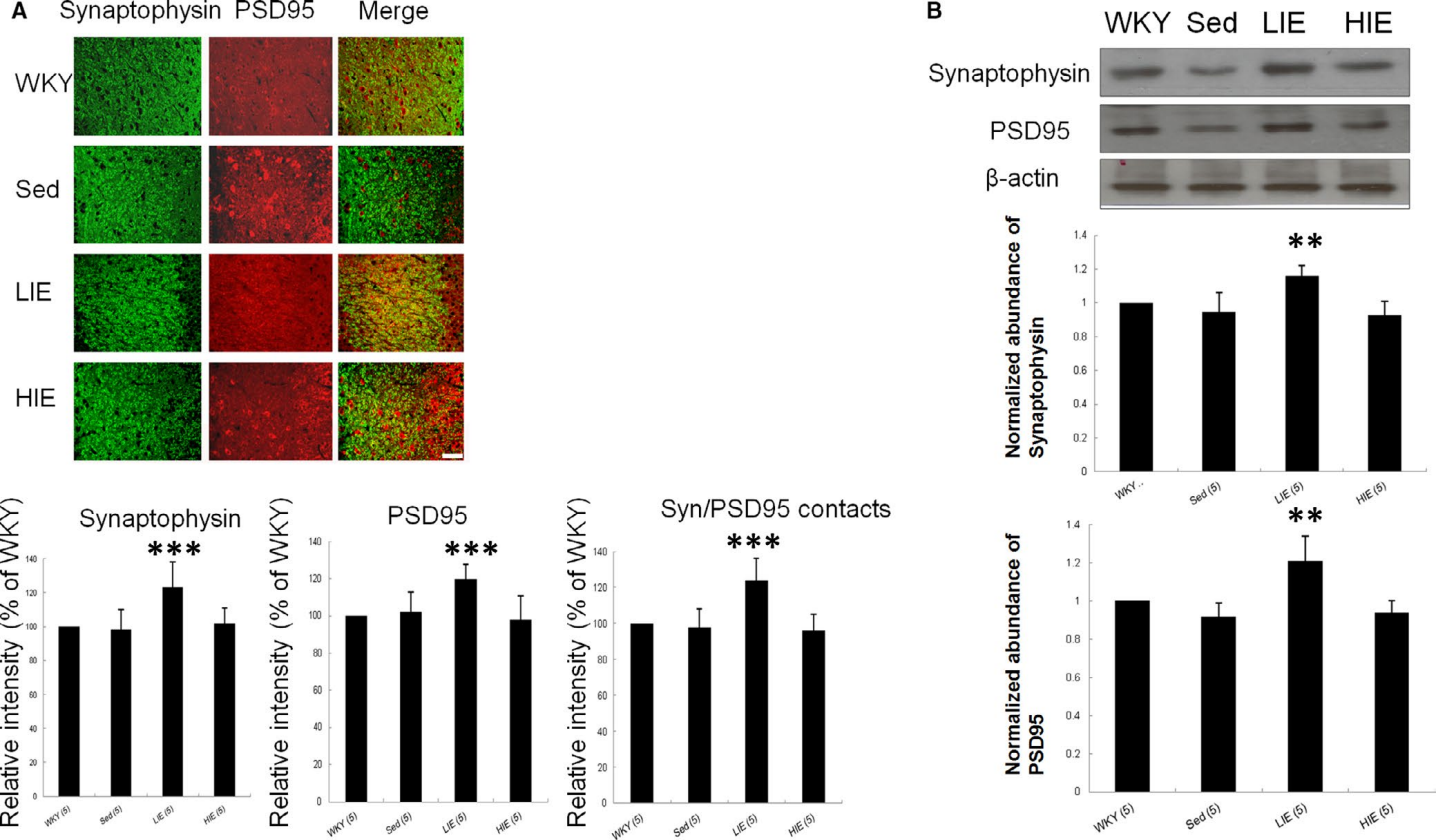
Synaptic density. (A) Presynaptic synaptophysin colocalizes with postsynaptic PSD95 in hippocampal dentate gyrus region. Analysis of the relative intensity of synaptophysin, PSD95 and SYN/PSD95 contacts. Quantitative data for synaptophysin and PSD95 show a significantly increased number of synaptophysin and PSD95 stained puncta in the LIE. Importantly, the total number of synaptophysin/PSD contacts (synaptic density) was also increased in the LIE. (B) Western blot confirmation for synaptophysin and PSD95 expression in hippocampus. Quantitative analysis of Western blot analysis. Data are shown as mean ± SD. *n* = 5 for each group. Scale Bar = 50 μm. Data expressed as mean  ± SD and analysed by one‐way ANOVA followed by Bonferroni's *post hoc* test. **p < 0.001, ***p < 0.001 versus WKY and sed SHR

We then investigated whether exercise resulted in changes in the number of synapses. Immunohistochemical staining for synaptophysin, a presynaptic marker, and PSD95, a postsynaptic marker, was conducted in the hippocampal dentate gyrus region (Figure 4A) to visualize presynaptic and postsynaptic specializations. Intensity of synaptophysin/PSD95 contacts was analysed as an index of reactive synaptic density, which displayed a 33.1% ± 3.1% increase in the hippocampal dentate gyrus region (*p *< 0.001) of LIE SHR compared with sedentary SHR. Western blot analysis confirmed a significant increase in both synaptophysin and PSD95 protein levels in the hippocampus of LIE SHR compared with sedentary SHR (Figure 4B). Thus, exercise resulted in a significant change in protein levels for both markers in the hippocampus.

### 3.4 Effects of hypertension and exercise intensity on cognitive function

**FIGURE 5:**
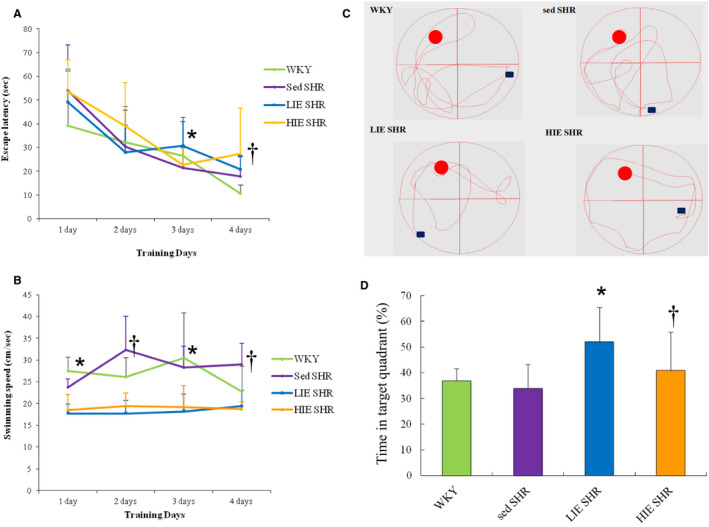
Performance differences of hypertension and exercise intensity in spatial learning and memory in the MWM test. (A) Escape latency on each training day. All groups showed improvements in finding the platform with repeated training. *p < 0.05 versus sed SHR at the same training day; ^†^ *p* < 0.05 versus WKY at the same training day. (B) Mean swimming speed in each training day. *p < 0.05 versus LIE and HIE SHR at the same training day; ^†^ *p* < 0.05 versus LIE and HIE SHR at the same training day. (C) Representative swims path during probe trial. (D) A probe test was used to determine spatial memory by measuring the percentage of the total time spent in the target quadrant. The LIE group rats showed improved memory compared with sed and HIE SHR groups, indicated by significant increased time swimming in the target quadrant. Data expressed as mean  ± SD (*n*  =  6) and analysed by two‐way ANOVA followed by Bonferroni's *post hoc* test. *p < 0.05 versus WKY and sed SHR; ^†^ *p* < 0.05 versus LIE SHR

The escape latencies of all of the rats gradually decreased (Figure 5A) In the Morris navigation task, indicating that the rats could learn to locate the submerged platform. There were no significant differences in escape latencies between WKY and sedentary SHR. Analyses of the swim speed have shown that there were no statistically throughout the training days between WKY and sedentary SHR (Figure 5B). However, the exercised rats, either in LIE or HIE, showed significantly slower speed compared with the sedentary rats. The exercised rats revealed more frequent immobility and floating visually compared with the sedentary rats.

The significant increase in the percentage of time spent in the correct quadrant confirmed the cognitive benefits of LIE in the probe test session (Figure 5C). In the probe trial, compared with normotensive WKY, sedentary SHR showed similar time in target quadrant (Figure 5D). Sedentary SHR spent about 34.0% ± 9.1% of the swim time in the target quadrant. LIE rats showed increased spatial location of exercised rats in the target quadrant and platform region (52.1% ± 13.4%), reinforcing that exercise improved the retrieval of spatial memory. However, the HIE group stayed less time in the target quadrant compared to the LIE (40.9% ± 14.9% vs. 52.1% ± 13.4% in LIE, *p* < 0.05). Taken together, these data suggested that the LIE, not HIE, ameliorated the learning and memory impairment.

## 4 DISCUSSIONS

To the best of our knowledge, this is one of very few studies to investigate the effect of different exercise intensities on cognitive function in chronically hypertensive animals. Our results showed that that exercise training, initiated at an established stage of hypertension, is capable of improving cognitive function. Although LIE (30 min’ swimming per session, five times per week, for 4 weeks) is not the effective way to lose weight, LIE definitely did not mean it was a waste of time; however, it is linked to improved overall cognitive function. Besides, this result suggests that the side effect of a HIE may counteract the beneficial effect of a LIE. Thus, adverse effects may occur if the exercise protocols are not applied appropriately. Our results indicated that LIE but not HIE improved memory consolidation in the rats, supporting an inverted U‐shaped dose‐response curve of the effect of exercise on memory consolidation. Furthermore, our results demonstrated that the SHR did not exhibit impaired learning in the water maze task, which is different to the findings reported in previous animal and clinical studies which indicated that chronic hypertension could impair learning and memory. Indeed, our results were consistent with the findings of Kadish et al.^35^ showing that learning impairment of SHR might not be entirely explained by hypertension. Finally, exercise improves cognitive function and causes structural and biochemical adaptation via modulating ROS‐synaptophysin pathways. Redox tuning has been shown to modulate the proliferation of neuronal progenitor cells. Our results were consistent with the notion of the bell‐shaped dose response between ROS and neurogenesis.^36^

Our results were consistent with the beneficial effects of appropriate exercise on cognitive function, as documented structurally by DHE stain, nitrotyrosine stain, Nissl stain, Golgi stain and immunofluorescent stain; molecularly by hippocampal synaptophysin and PSD95 proteins; biochemically by plasma superoxide levels and tissue MDA levels, and functionally by Morris water maze test. Our conclusions are supported by 3 lines of evidence.

### 4.1 Hypertension

Our results showed that chronic hypertension did not impair spatial learning and memory impairment. In comparison to WKY rats, sedentary SHRs did not have any impairment in spatial working memory and reference memory. The chronic hypertension in the SHR rats does not compromise motor behaviour. The rats of WKY and sedentary SHR swam at similar speeds.

Although some studies have demonstrated an association between hypertension and cognitive loss, there were controversial results regarding the association. Possible explanations for this inconsistency include heterogeneity in duration of follow‐up, the measure methods of cognitive function and sample size. First, it appears that long duration of hypertension may be required for the adverse cognitive effects. For hypertension to induce neurodegeneration, more than 1–1.5 decades of uncontrolled hypertension may be required.^37^ The SHR model used in this study was 28‐week‐old, which is not representative of the advanced hypertensive period.^38^ Compared with WKY, SHRs older than 32 weeks of age exhibited a significant increase in ROS in the hippocampus.^38^ Second, as many neuropsychological tests are available, many different instruments were used in these studies to evaluate different cognitive domains. Therefore, the discrepancies may have been caused by differences in the tasks used to assess learning and memory. For example, a radial maze involves navigating predetermined constrained routes, whereas a water maze requires the animal to navigate unconstrained routes with the water acting as a negative stimulus. As the sensory cues in these tests are different (water vs. air), the sensory inputs would also be different, and thus, the learning and memory tasks required for the two tests may also be different. Indeed, previous studies have shown that behavioural parameters detected in the water maze test were highly correlated with the anatomical changes of the dentate gyrus.^39^ Finally, a relatively small sample size with 6 rats in each group was assessed. Thus, a type II error cannot be ruled out.

### 4.2 Exercise intensity and ROS

The effects of exercise intensity on the cognitive function may be affected. Few studies have investigated whether regular exercise above a certain intensity could cause harm, and hence, the optimal exercise load required to enhance physiological function, such as brain function is unknown. Our results demonstrate that low‐intensity, not high‐intensity, exercise improved cognitive functions in SHR. Exercise intensity would differentially affect susceptibility of rat hippocampal lesions.^40^

The effect of exercise on the activities of ROS is complex. The intensity of exercise has been reported to significantly affect outcomes, and exhaustive training has been suggested to potentially worsen oxidative stress, thereby making the brain more susceptible to ischaemic insults.^41^ Low‐intensity swimming training has been shown to enhance the antioxidant defence system, whereas HIE has not demonstrated this protective effect.^42^ These findings clearly demonstrate a U‐shaped relationship between the intensity of physical activity and neuroprotection.

We suggest that excessively high levels of ROS can impair brain cell function by exceeding the adaptive cell tolerance, leading to significant oxidative damage, apoptosis and necrosis. Our results are consistent with those of Powers et al.^43^ showing that the overproduction of ROS induced by exhaustive exercise training can lead to oxidative stress and related tissue damage. However, Ogonovszky et al.^44^ have shown that rats were subjected to moderate‐, strenuous‐ and over‐training and found no increased ROS accumulation assessed by reactive carbonyl derivative even with strenuous training and over‐training. The reason for this discrepancy between our findings and theirs is unclear. However, it can be explained, at least in part, the differences in the protocol and target molecules.

### 4.3 Synapse dynamics underlie cognitive plasticity

Exercise induced neuronal networks by regulating dendritic arborization and synaptic density. Our study showed that exercise increased dendritic complexity and the number of dendritic spines in the dentate gyrus. ROS are key mediators of blood‐brain barrier breakdown to increase the barrier permeability.^45^ A key effect of ROS is lipid peroxidation, and increasing evidence suggests that higher levels of lipid peroxidation and/or its metabolites may be neurotoxic.^46^ This is one of the first studies to measure the content of synapse dynamics with different exercise loads. We found that the levels of synaptophysin and PSD95 were increased by LIE. PSD95 is a key molecule in mature synapses.^47^ Previous studies have shown that targeted disruption of PSD95 exhibited severe deficits in spatial learning and altered activity‐dependent synaptic plasticity in the hippocampus, especially dentate gyrus synapses.^47^ Since synaptophysin and PSD95 regulated neuronal synaptic activity,^48^ increases in synapse number assessed by the intensity of synaptophysin puncta colocalized with PSD95 can lead to cognitive improvement. Our results are consistent with the notion that increased expression of synaptophysin and PSD95 was related to improved cognitive memory due to enhanced synaptic plasticity.

We assessed whether animals are equal in their ability to swim and motivation to escape by assessing swimming speed during learning. We found there was a significant difference of swimming speed between sedentary and exercised groups because the exercised rats had more time to be floating during the training trial and the probe trial. After initially spending periods swimming and attempting to escape, most of the rats stopped swimming and floated in the water. This immobility has been described as ‘behavioural despair’ or ‘learned helplessness’, both of which are associated with depression. In addition, this immobility has been suggested to be due to the rats being incapable or unwilling to continue making an effort rather than generalized hypoactivity. Assuming that immobility is a learned process, the rats may have learned that it is best to wait passively to be removed from the water.^49^, ^50^ Another study suggested that learning to become immobile may be an adaptive response, as it conserves the energy needed to prolong survival.^51^ The acquisition of immobility represents an adaptive cognitive process. Thus, the exercised rats may try to preserve the energy by more time to be floating.

Our study suggests that LIE was able to improve the cognitive function. The mechanisms by which exercise improved cognitive function remain to be defined. However, several factors can be excluded. (1) Systolic blood pressure: the exercise‐induced improvement in cognition does not seem to be due to the decrease in systolic blood pressure, since systolic blood pressure was similar among the sedentary and exercised SHR groups. The effects of exercise on blood pressure in SHR vary; some studies have reported that exercise has an anti‐hypertensive effect,^52^ while others have reported no change in hypertension,^53^ and others have even reported exacerbations.^54^ Our results are consistent with previous studies, in that swimming did not reduce blood pressure in the SHR with established hypertension.^54^ Besides, previous studies have shown that elevated pulse pressure was associated with age‐related cognitive decline.^55^ Given similar systolic blood pressure and pulse pressure after exercise training, it is unlikely that pulse pressure played a role in improving cognitive function. Thus, exercise improved cognitive function, independently of haemodynamics changes. (2) Body weight: as usually seen in literature, our SHR presented lower body weight than the WKY group.^56^ The LIE protocol did not change body weight. Although previous studies have shown that body weight affected cognitive function,^57^ the improvement of cognitive function in LIE obviously not related to body weight.

## 5 CLINICAL IMPLICATIONS

Understanding the cellular mechanisms responsible for improvements in brain function caused by physical exercise will help to improve the understanding of cognition in general and also to identify mechanisms that could be used to develop therapy to improve learning and memory. The implication for humans is that physical exercise provides both mental stimulation and also improvements in the cognitive domain of spatial learning.

This study has important clinical implications. Older patients are usually reluctant to take part in exercise programmes, especially high‐intensity programmes. Our results suggest that older people with established hypertension can still benefit clinically from low‐intensity aerobic physical exercise. Furthermore, LIE may lead to better adherence than HIE. Understanding the utility of reduced intensity alternative exercise programmes is important to provide tailored clinical exercise interventions and help prevent side effects and treatment failure, and enhance efficacy.

## 6 STUDY LIMITATIONS

A number of important limitations of the present study need to be considered. Previous studies have shown that short‐term HIE programmes can increase lipid peroxidation caused by oxidative stress^58^ and that long periods of exercise can ameliorate the lipid peroxidation process to prevent tissue damage.^59^ Thus, the negative cognitive protection in one‐month HIE cannot be extended into the long duration of exercise. Furthermore, we only investigated the effects of two different exercise intensities, and this may not be sufficient to clearly delineate the effect of exercise intensity in SHR. Consequently, we cannot definitively conclude that only LIE reduces cognitive deficits. Further studies including different intensities of exercise are warranted to provide more accurate and reliable assessments.

## 7 CONCLUSIONS

The present data show that physical exercise is considered a complementary therapy in the management of the cognitive decline through the regulation of ROS‐dependent synaptic plasticity in the hippocampus. Further clinical studies are urgently needed to determine the safety and efficacy of LIE in hypertensive patients in cognitive functions.

## CONFLICT OF INTEREST

The authors declare no conflict of interest.

## AUTHOR CONTRIBUTIONS

**Cheng‐Che Lee:** Methodology (supporting); Project administration (supporting); Writing‐original draft (supporting); Writing‐review & editing (supporting). **De‐Yu Wu:** Methodology (supporting); Project administration (supporting); Writing‐original draft (supporting); Writing‐review & editing (supporting). **Syue‐yi Chen:** Conceptualization (supporting); Data curation (lead); Formal analysis (lead); Methodology (lead); Project administration (lead); Writing‐review & editing (supporting). **Yi‐Pin Lin:** Conceptualization (supporting); Investigation (supporting); Writing‐original draft (supporting); Writing‐review & editing (supporting). **Tsung‐Ming Lee:** Conceptualization (lead); Data curation (supporting); Formal analysis (supporting); Funding acquisition (lead); Resources (lead); Supervision (lead); Writing‐review & editing (supporting).
